# Supplementary material for: Plastic roles of pericytes in the blood–retinal barrier
Source: Nat Commun. 2017 May 16;8:15296. doi: 10.1038/ncomms15296 (PMC5440855; doi:10.1038/ncomms15296)
Supplement: Supplementary Information — Supplementary Figures and Supplementary Tables. [file ncomms15296-s1.pdf]

Supplementary Figure 1

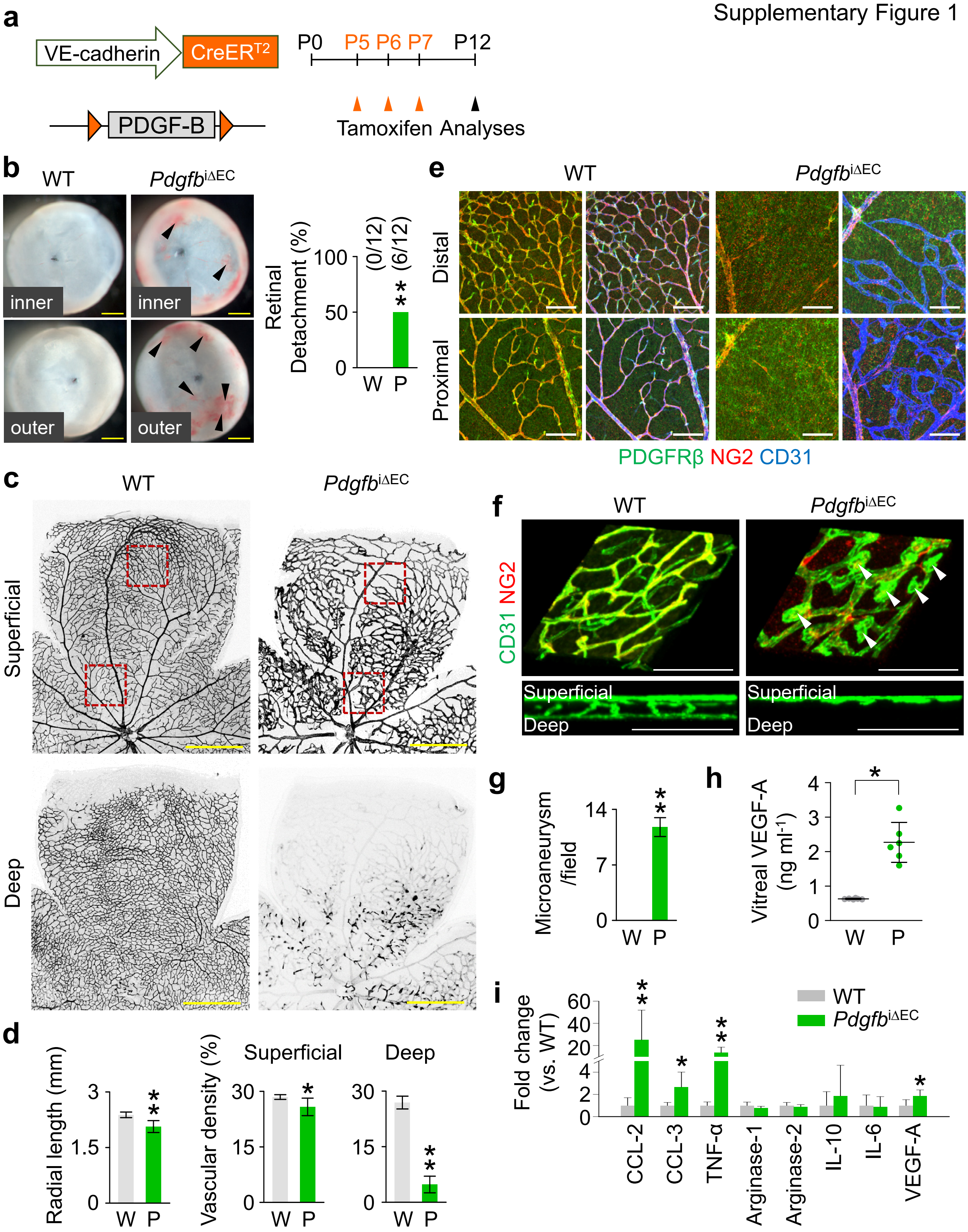

Supplementary Figure 1. Pericyte coverage is critical for formation and maturation of BRB formation during the postnatal period. (**a**) Diagram depicting the experiment schedule for EC-specific depletion of PDGF-B in retinal vessels from P5 and their analyses at P12 using *Pdgfb*$^{i\Delta EC}$ mice. (**b**) Images of inner and outer surfaces of retinal cup, and comparison of frequency of retinal detachment in WT (W; *n* = 12) and *Pdgfb*$^{i\Delta EC}$ (P; *n* = 12) mice. Multiple retinal hemorrhages (arrowheads) are observed in *Pdgfb*$^{i\Delta EC}$ mice. (**c**) Images of CD31$^+$ superficial and deep vascular plexus in WT and *Pdgfb*$^{i\Delta EC}$ mice. Severe impairment of deep vascular plexus formation is detected in *Pdgfb*$^{i\Delta EC}$ mice. (**d**) Comparisons of indicated parameters in WT (W; *n* = 6) and *Pdgfb*$^{i\Delta EC}$ (P; *n* = 6) mice. (**e**) Distribution of PDGFRβ$^+$ or NG2$^+$ pericytes onto CD31$^+$ retinal vessels in boxed regions of (**c**). Note that PDGFRβ$^+$ pericytes and NG2$^+$ pericytes are almost identical, and they tightly cover all vessels in retina of WT mice, while their coverage onto vessels are severely impaired in both proximal and distal zones of retina in *Pdgfb*$^{i\Delta EC}$ mice. (**f**) 3D images of CD31$^+$ vascular plexus in WT and *Pdgfb*$^{i\Delta EC}$ mice. Multiple microaneurysm (arrowheads) but no vertical branch and deep vascular plexus are observed in *Pdgfb*$^{i\Delta EC}$ mice. Note the ballooning of CD31$^+$ vessels with inadequate percitye coverage, which fails to sprout into deep retinal layer and leads to microaneurysm formation. (**g-i**) Comparisons of number of microaneurym, VEGF-A level in vitreous fluid of eye, and gene expression profiles in retinas of WT (W; *n* = 6) and *Pdgfb*$^{i\Delta EC}$ (P; *n* = 6) mice. All error bars represent mean ± s.d. *P < 0.05, **P < 0.01 versus WT by Mann-Whitney *U* test. Scale bars, 100 μm (white) and 500 μm (yellow).

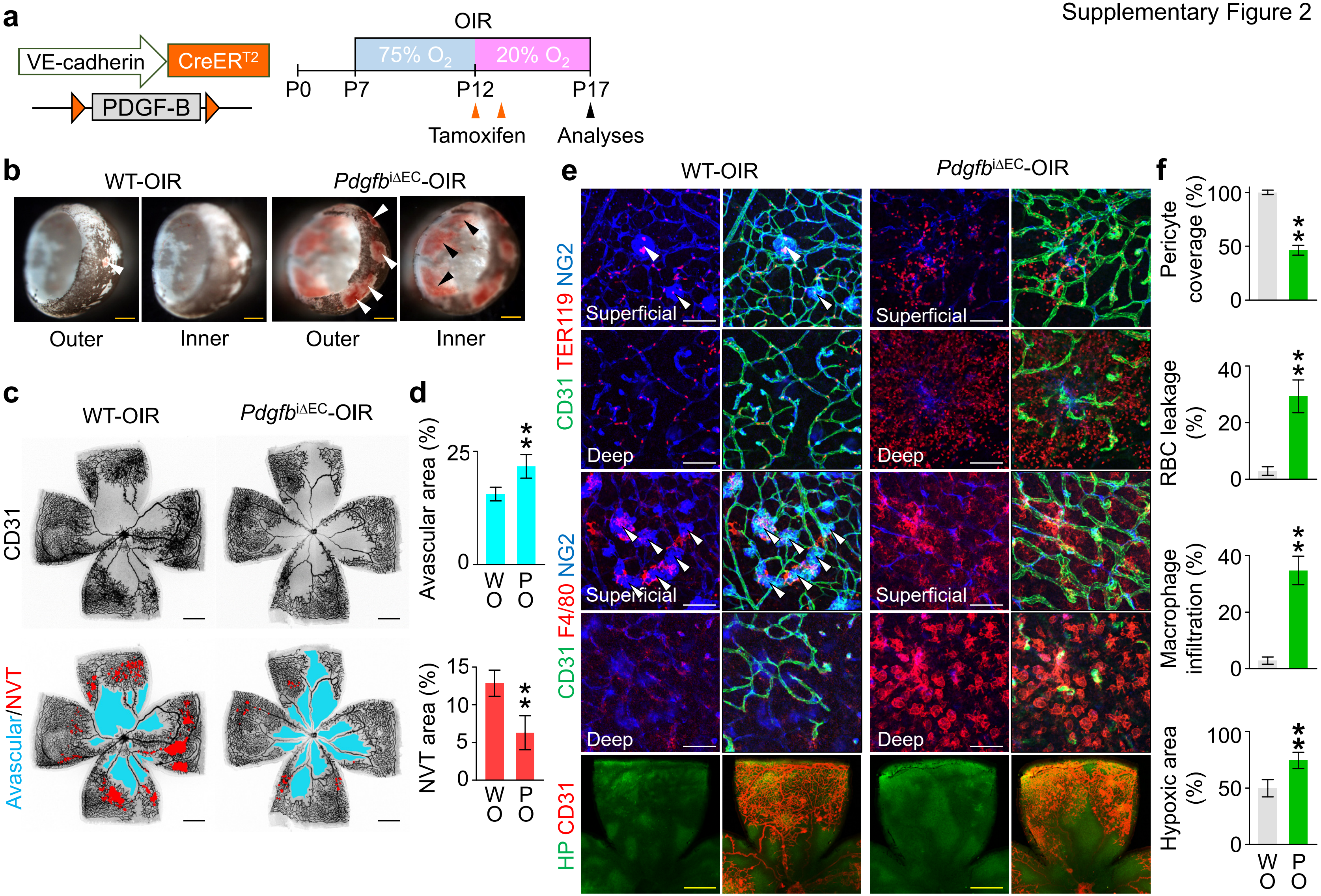

Supplementary Figure 2. Pericyte detachment exacerbates vascular impairments in OIR mouse model. (**a**) Diagram depicting the experiment schedule for PDGF-B depletion in retinal ECs and generation of OIR model in *Pdgfb*$^{i\Delta EC}$ mice (*Pdgfb*$^{i\Delta EC}$-OIR). (**b**) Images of inner and outer surfaces of retinal cup of WT-OIR and *Pdgfb*$^{i\Delta EC}$-OIR mice. Arrowheads indicate hemorrhages. (**c,d**) Images and comparisons of CD31^{+} vessels, avascular area (blue), and neovascular tuft (NVT) area (red) in WT-OIR (WO; *n* = 6) and *Pdgfb*$^{i\Delta EC}$-OIR (PO; *n* = 6) mice. (**e**) Images of retinal vascular phenotypes between WT-OIR and *Pdgfb*$^{i\Delta EC}$-OIR mice. CD31^{+} vessels, NG2^{+} pericyte coverage, TER119^{+} RBC leakage, F4/80^{+} macrophage infiltration and hypoxic area in superficial and deep retinal layers are shown. Arrowheads indicate NVTs densely covered with NG2^{+} pericytes in WT-OIR mice. (**f**) Comparisons of indicated parameters in WT-OIR (WO; *n* = 6) and *Pdgfb*$^{i\Delta EC}$-OIR (PO; *n* = 6) mice. Error bars represent mean ± s.d. $^{**}P < 0.01$ versus WO by Mann-Whitney *U* test. Scale bars, 100 μm (white) and 500 μm (black, yellow).

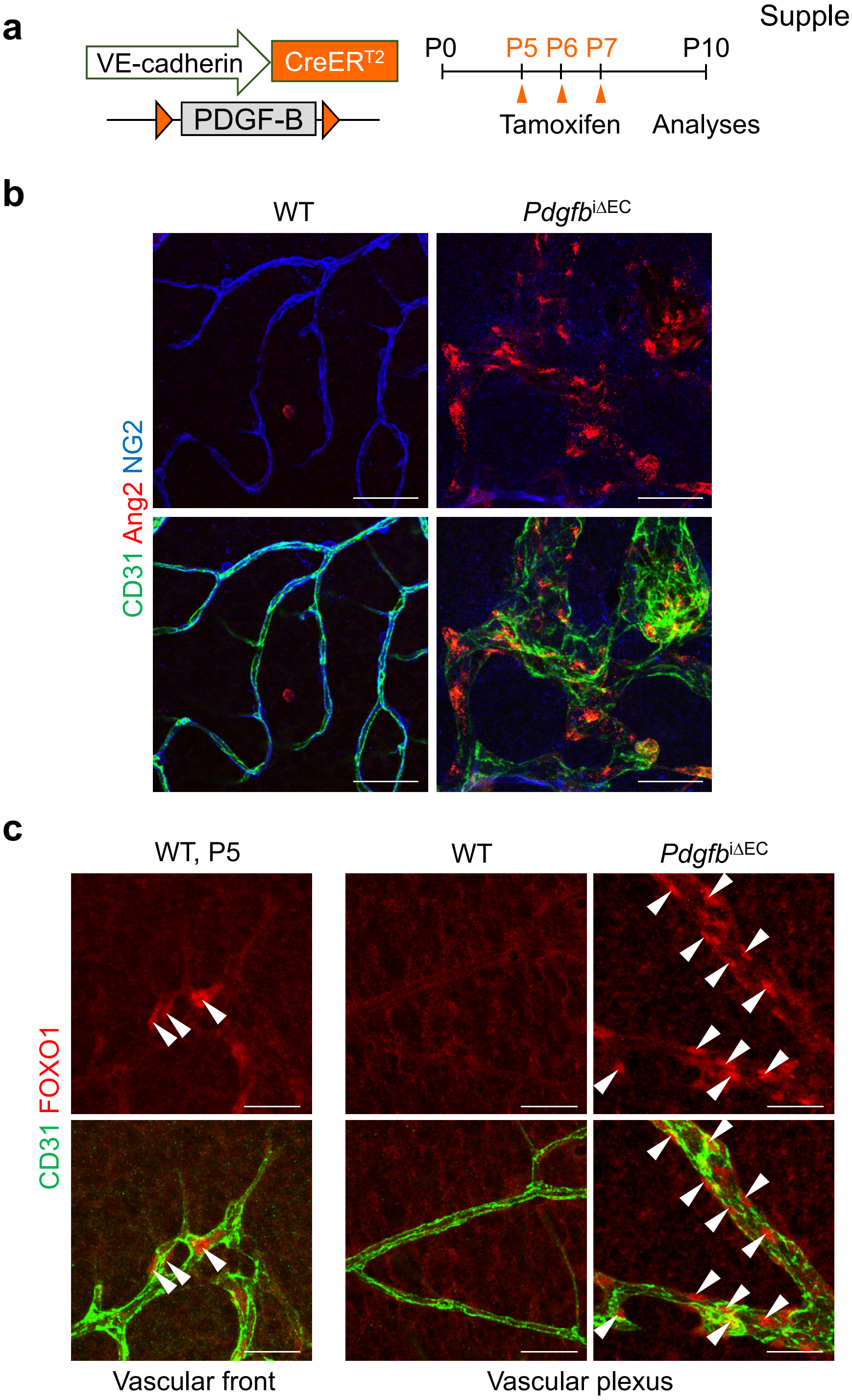

Supplementary Figure 3. Nuclear localization of FOXO1 and high expression of Ang2 in the pericyte-uncovered retinal vessels in the *PDGF-B*$^{i\Delta EC}$ mice. (**a**) Diagram depicting the experiment schedule for EC-specific deletion of PDGF-B in retinal vessels from P5 and their analysis at P10 in *Pdgfb*$^{i\Delta EC}$ mice. (**b**) High magnification images of Ang2 expression in CD31^{+} vessels in WT and *Pdgfb*$^{i\Delta EC}$ mice. *Pdgfb*$^{i\Delta EC}$ mice show strong Ang2 signals with several dots clustered within the retinal vessels. (**c**) High magnification images of FOXO1 expressions in CD31^{+} vessels in WT and *Pdgfb*$^{i\Delta EC}$ mice. Round and condensed signals of FOXO1 (arrowheads) are observed in the vascular front of the retinal vessels in WT mice and in the retinal vessels in *Pdgfb*$^{i\Delta EC}$ mice. All scale bars, 50 μm (white).

Supplementary Figure 4

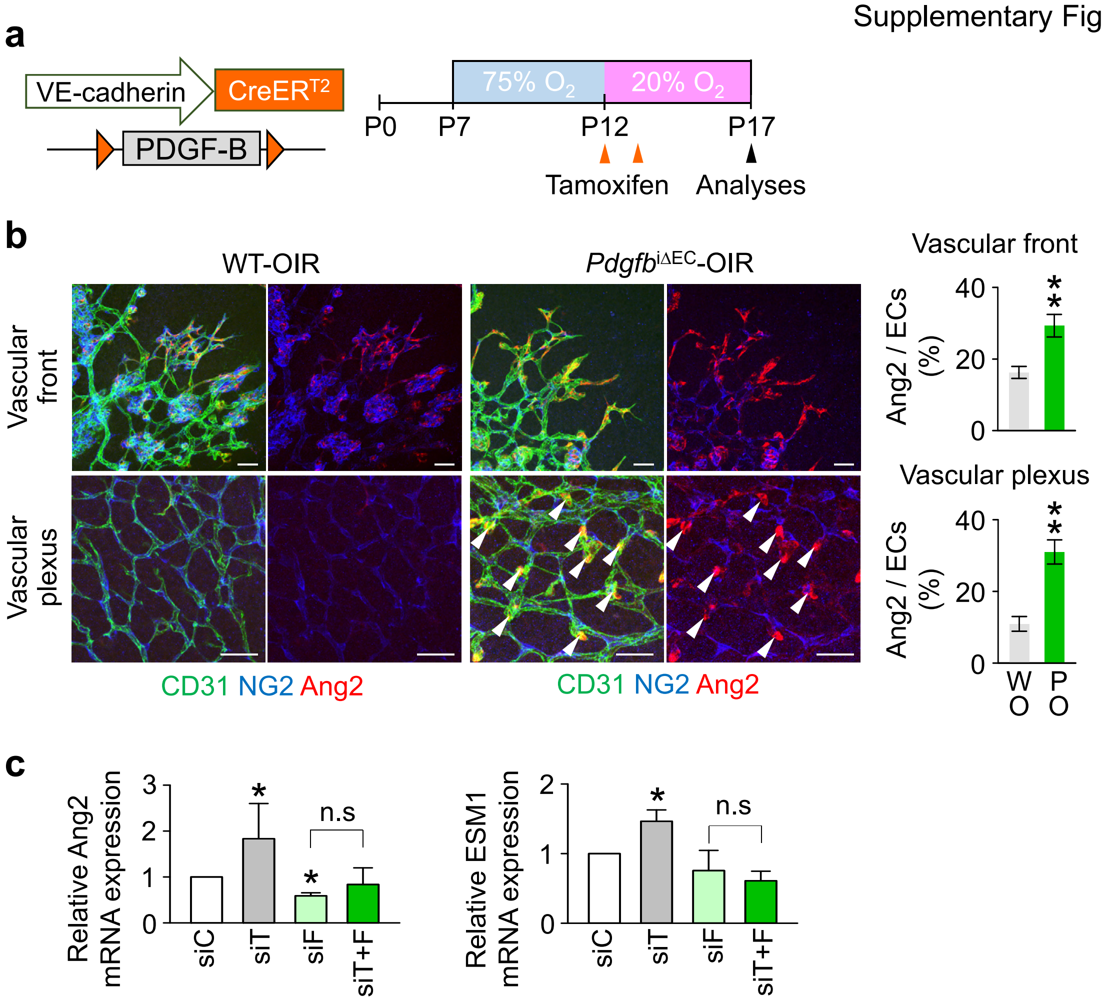

Supplementary Figure 4. Abundant expression of Ang2 by pericyte-detached retinal vessels at vascular front and microaneurysmal lesions of OIR mouse model. (**a**) Diagram depicting the experiment schedule for timing of PDGF-B depletion in retinal ECs and generation of OIR model in *Pdgfb*$^{i\Delta EC}$ mice (*Pdgfb*$^{i\Delta EC}$-OIR). (**b**) Images and comparisons of Ang2 expression in vascular front and plexus areas of WT-OIR (WO; *n* = 6) and *Pdgfb*$^{i\Delta EC}$-OIR (PO; *n* = 6) mice. Strong expression of Ang2 by ECs is observed at vascular front and microaneurysm lesions (arrowheads) that are not covered by NG2^{+} pericytes in *Pdgfb*$^{i\Delta EC}$-OIR mice. Error bars represent mean ± s.d. ***P* < 0.01 versus WO by Mann-Whitney *U* test. All scale bars, 100 μm. (**c**) Changes of Ang2 and ESM1 mRNA levels in primary cultured HUVECs transfected with control siRNA (siC), Tie2 siRNA (siT), FOXO1 siRNA (siF), and siT plus siF (siT+F). Each group, *n* = 6. Error bars represent mean ± s.d. **P* < 0.05 versus siC; n.s, non-significant versus siF by Kruskal-Wallis test.

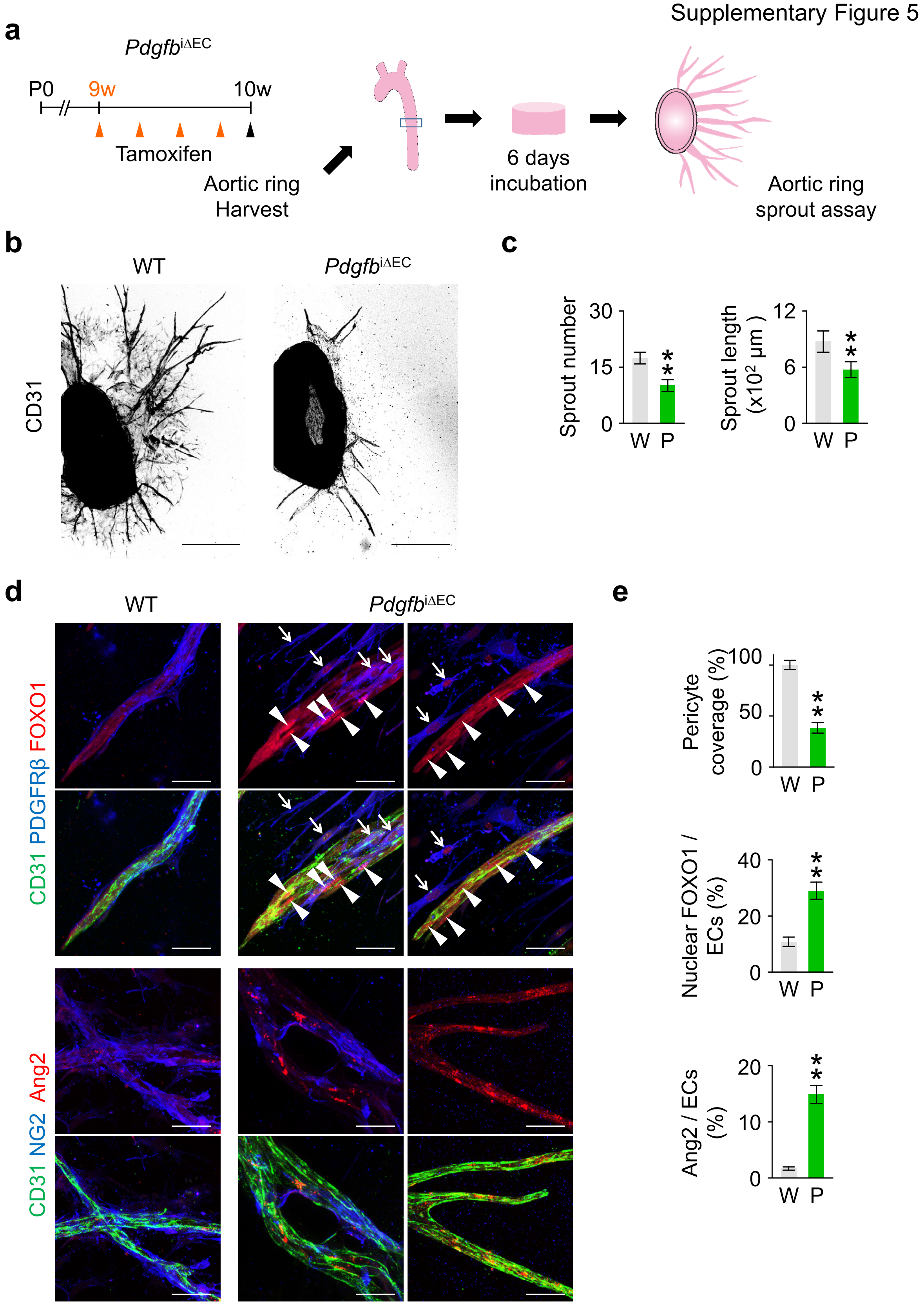

Supplementary Figure 5. Impaired interaction between ECs and pericytes induces FOXO1 activation and Ang2 expression in aortic ring assay. (**a**) Diagram for EC-specific depletion of PDGF-B in 9-week-old *Pdgfb*$^{i\Delta EC}$ mice and aortic ring harvest 1 week later. Analyses of sprouting vessels from aortic ring were performed after 6 days of incubation after the harvest. (**b-c**) Images and comparison of CD31^{+} sprouting vessels with the indicated parameters in the aortic ring of WT (W, n = 6) and *Pdgfb*$^{i\Delta EC}$ (P, n = 6) mice. (**d**) High magnification images showing the interaction between PDGFRβ^{+} or NG2^{+} cells and CD31^{+} ECs and expression and localization of FOXO1 and Ang2 in the vessels sprouting from the aortic ring of W and P mice. Detachment of PDGFRβ^{+} cells (arrows) from CD31^{+} ECs together with nuclear-localized FOXO1 (arrowheads) and high expression of Ang2 in ECs were observed in the P group. (**e**) Comparisons of indicated parameters in W (n = 6) and P (n = 6). Error bars represent mean ± s.d. $^{**}P < 0.01$ versus Error bars represent mean ± s.d. $^{*}P < 0.05$, $^{**}P < 0.01$ versus WT by Mann-Whitney *U* test. Scale bars, 500 μm (black) and 50 μm (white).

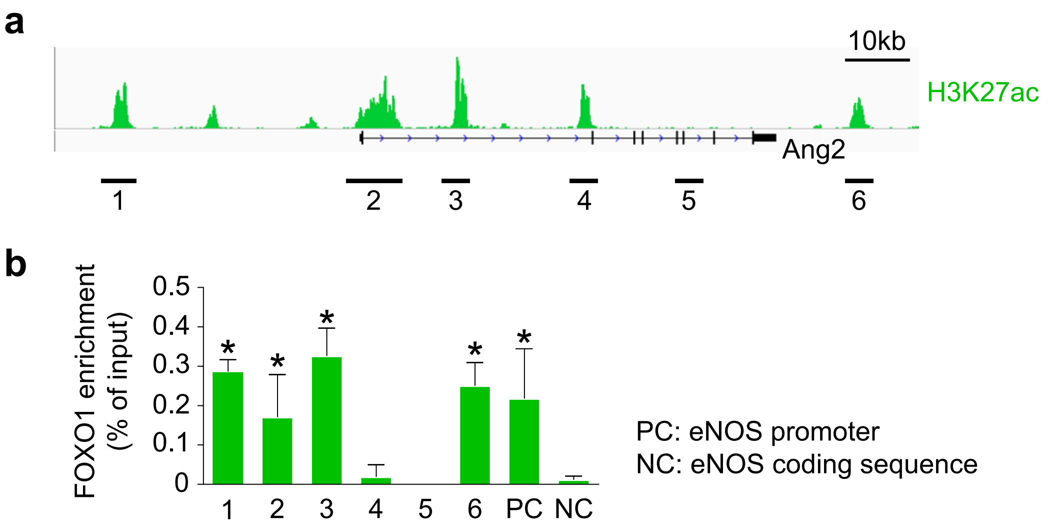

Supplementary Figure 6. Confirmation of FOXO1-induced transcriptional activation of Ang2 expression by ChIP-qPCR (**a**) H3K27ac ChIP-seq binding profile at Ang2 locus (GSM733691). Possible enhancers were selected based on the H3K27ac ChIP-seq data. Selected regions are indicated by black bars (1; upstream, 2; promoter, 3-5; Intron, 6; downstream). (**b**) ChIP-qPCR results showing FOXO1 enrichment over input (n = 4). Among enhancer candidates, FOXO1 binding is significantly enriched at the distal enhancer (1,6), promoter (2), and intronic enhancer (3) regions of Ang2. PC; positive control. NC; negative control. Error bars represent mean ± s.d. *P < 0.05, versus NC by Mann-Whitney U test.

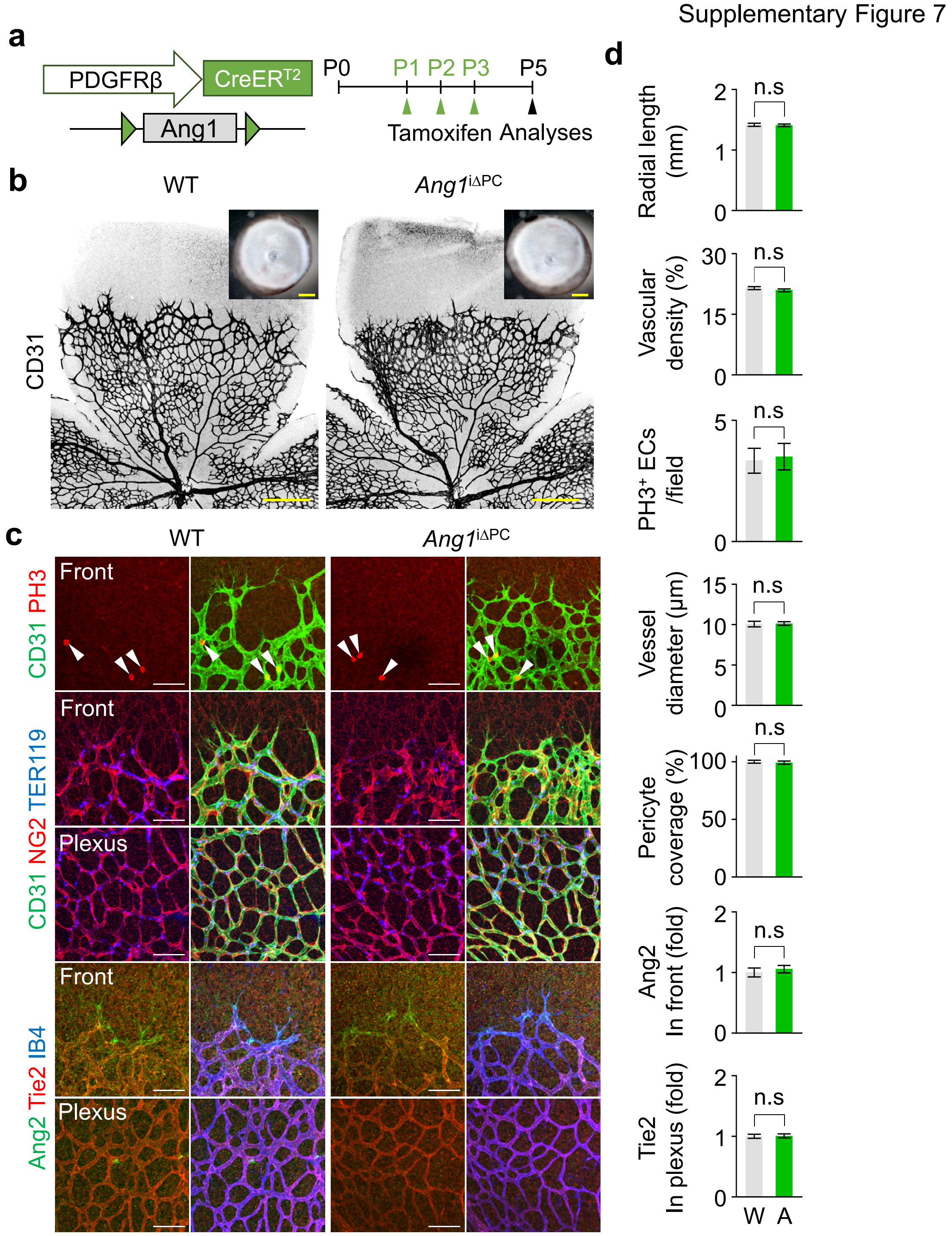

Supplementary Figure 7. Retinal pericytes do not produce Ang1 to regulate vascular growth and BRB maturation. (**a**) Diagram depicting the experiment schedule for pericyte-specific depletion of Ang1 in retinal vessels from P1 and their analyses at P5 using *Ang1*$^{i\Delta PC}$ mice. (**b,c**) Images of inner surface (right upper panels), CD31^{+} superficial vascular plexus, PH3^{+}/CD31^{+} proliferating ECs (arrowheads), NG2^{+} pericyte coverage, TER119^{+} RBC localization, and Tie2 and Ang2 expressions in the indicated zones of retinas of WT and *Ang1*$^{i\Delta PC}$ mice at P5. Similar findings are observed in each group (n = 6). (**d**) Comparisons of indicated parameters in WT (W; n = 6) and *Ang1*$^{i\Delta PC}$ (A; n = 6) mice. Error bars represent mean ± s.d. n.s versus WT by Mann-Whitney U test. Scale bars, 100 μm (white) and 500 μm (yellow).

Supplementary Figure 8

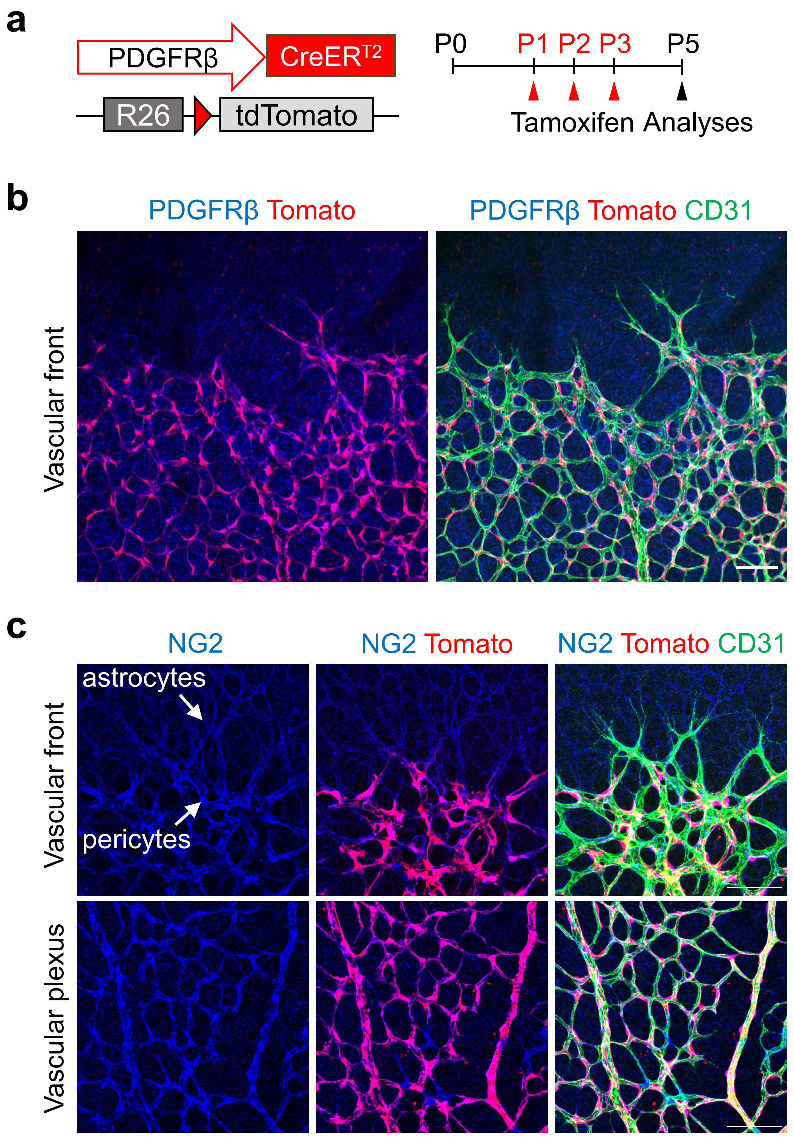

Supplementary Figure 8. High targeting efficiency for PDGFRβ$^+$ or NG2$^+$ pericytes in retinal vessels by cre-mediated recombination using PDGFRβ-Cre-ER^{T2} mice. (**a**) Diagram of gene construct of PDGFRβ-Cre-ER^{T2} mouse crossed with R26-tdTomato reporter mouse, and schedule for induction of cre-mediated recombination with tamoxifen. (**b,c**) Images of CD31$^+$ retinal vessels and PDGFRβ$^+$ or NG2$^+$ pericytes at vascular front and plexus in PDGFRβ-Cre-ER^{T2}:R26-tdTomato mice. tdTomato is expressed in most (> 95%) of PDGFRβ$^+$ or NG2$^+$ pericytes, while it is not expressed in NG2$^+$ astrocytes. All scale bars, 100 μm.

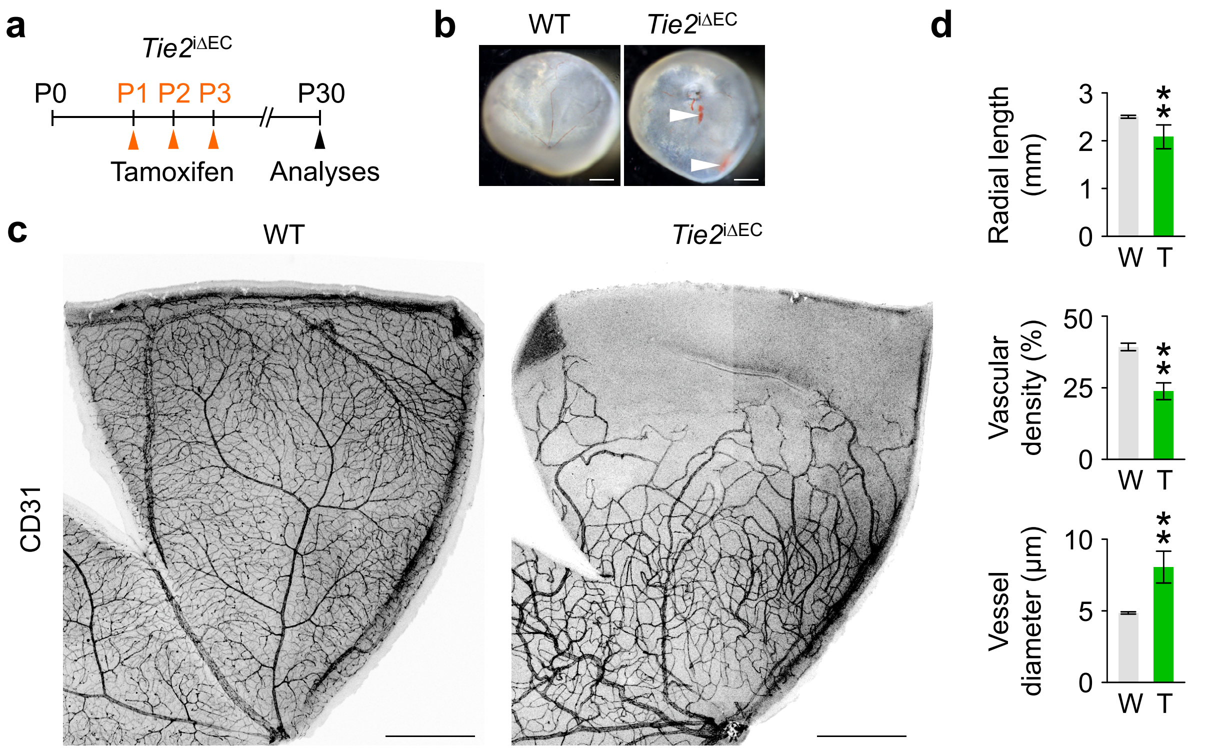

Supplementary Figure 9. Vascular impairments due to endothelial Tie2 depletion after birth continue until adulthood. (**a**) Diagram depicting the experiment schedule for EC-specific depletion of Tie2 in retinal vessels immediately after birth and their analyses at P30 in *Tie2*$^{i\Delta EC}$ mice. (**b-d**) Images of inner surface of retinal cup and CD31^{+} retinal vessels, and comparisons of indicated parameters in WT (W; *n* = 6) and *Tie2*$^{i\Delta EC}$ (T; *n* = 6) mice. Minor leakages (arrowheads) are observed in the retinal cup of the *Tie2*$^{i\Delta EC}$ mice. Error bars represent mean ± s.d. $^{**}P < 0.01$ versus WT by Mann-Whitney *U* test. Scale bars, 500 μm (white, black).

Supplementary Figure 10

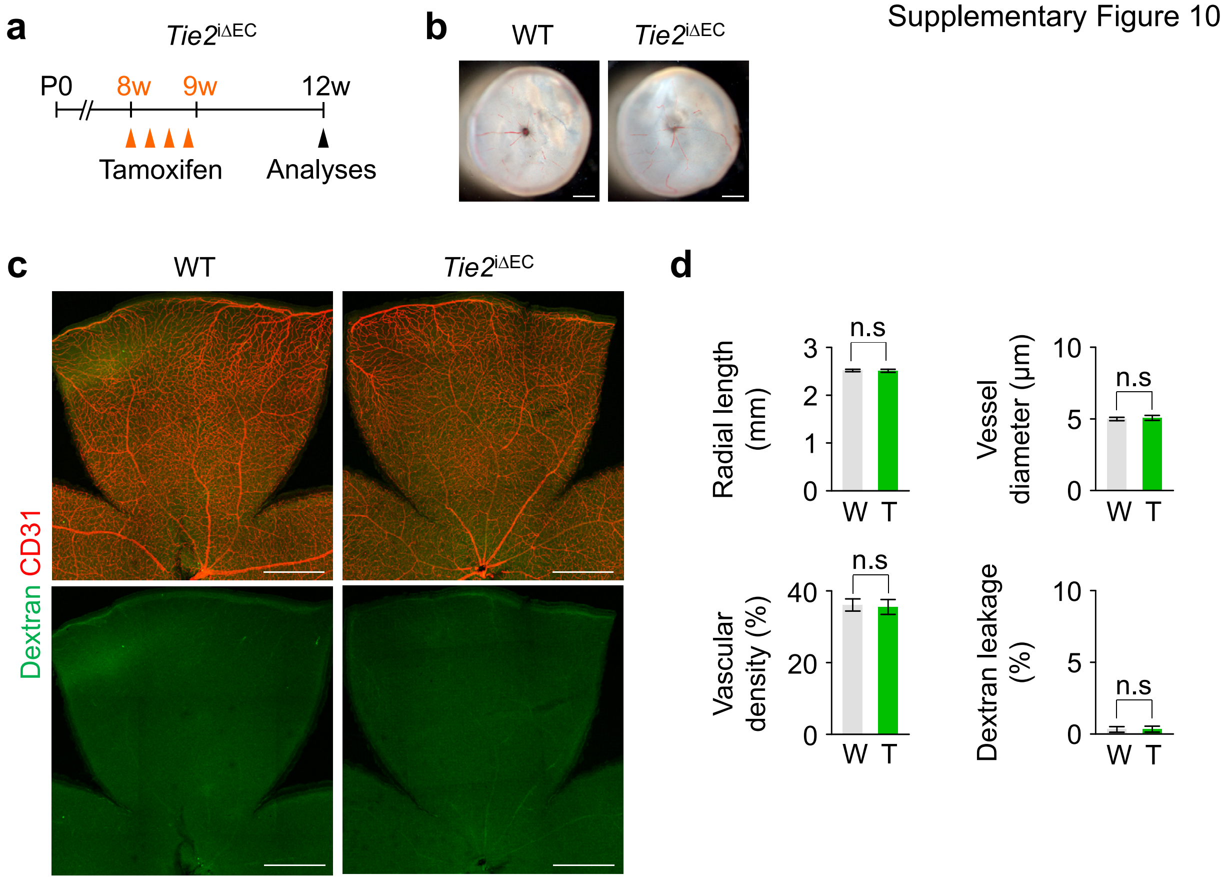

Supplementary Figure 10. Dispensable role of endothelial Tie2 during adulthood. (**a**) Diagram depicting the experiment schedule for EC-specific depletion of Tie2 in retinal vessels starting 8 weeks after birth and their analyses 4 weeks later using *Tie2*$^{i\Delta EC}$ mice. (**b-d**) Images of inner surface of retinal cup and CD31^{+} retinal vessels, and comparisons of indicated parameters in WT (W; *n* = 6) and *Tie2*$^{i\Delta EC}$ (T; *n* = 6) mice. Error bars represent mean ± s.d. n.s., non-significant versus WT by Mann-Whitney *U* test. All scale bars, 500 μm (white).

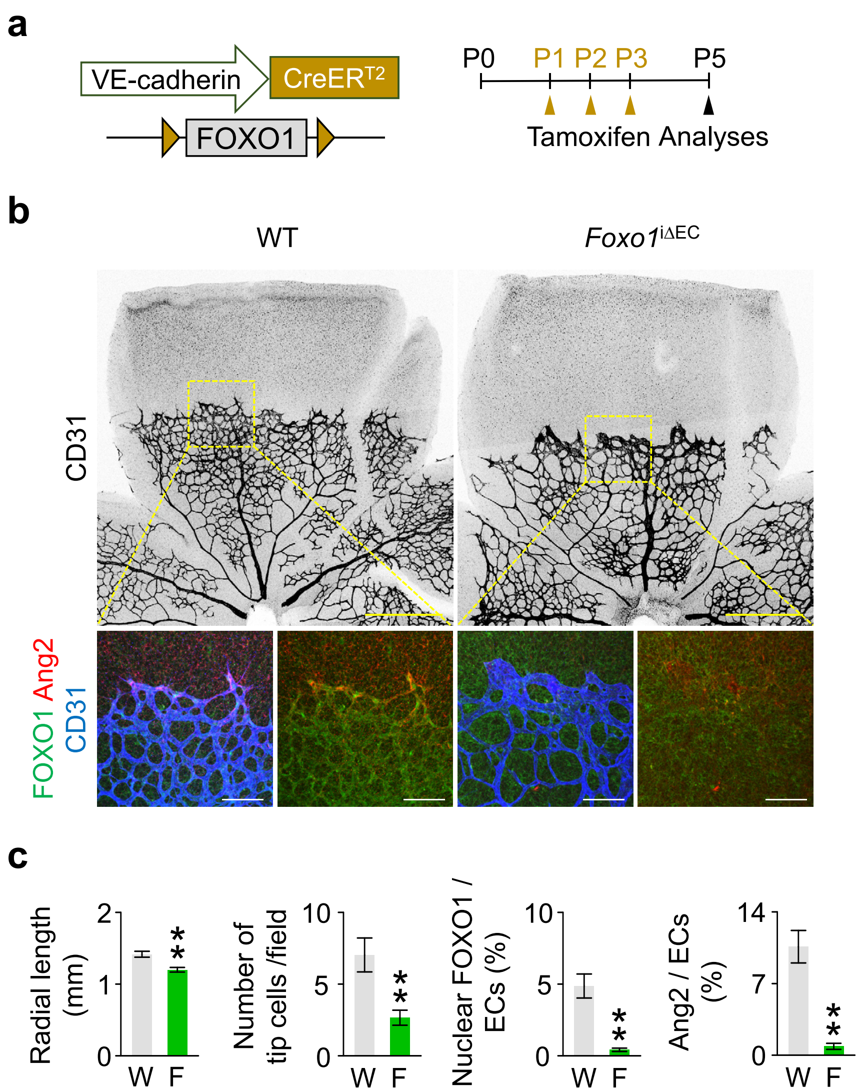

Supplementary Figure 11. Endothelial FOXO1 is critical for sprouting angiogenesis during early postnatal period. (**a**) Diagram depicting the experiment schedule for EC-specific depletion of FOXO1 in retinal vessels from P1 and their analyses at P5. (**b**) Images of CD31^{+} retinal vessels in WT and *Foxo1*$^{i\Delta EC}$ mice. Boxed areas in vascular front region (dotted-lines) are magnified in lower panels showing less sprouting and blunt tip ECs, and much less expressions of FOXO1 and Ang2 in retinal vessels of *Foxo1*$^{i\Delta EC}$ mice compared with those of WT mice. (**c**) Quantification of indicated parameters in WT (W; n = 6) and *Foxo1*$^{i\Delta EC}$ (F; n = 6) mice. Error bars represent mean ± s.d. **P < 0.01 versus WT by Mann-Whitney U test. Scale bars, 100 μm (white) and 500 μm (yellow).

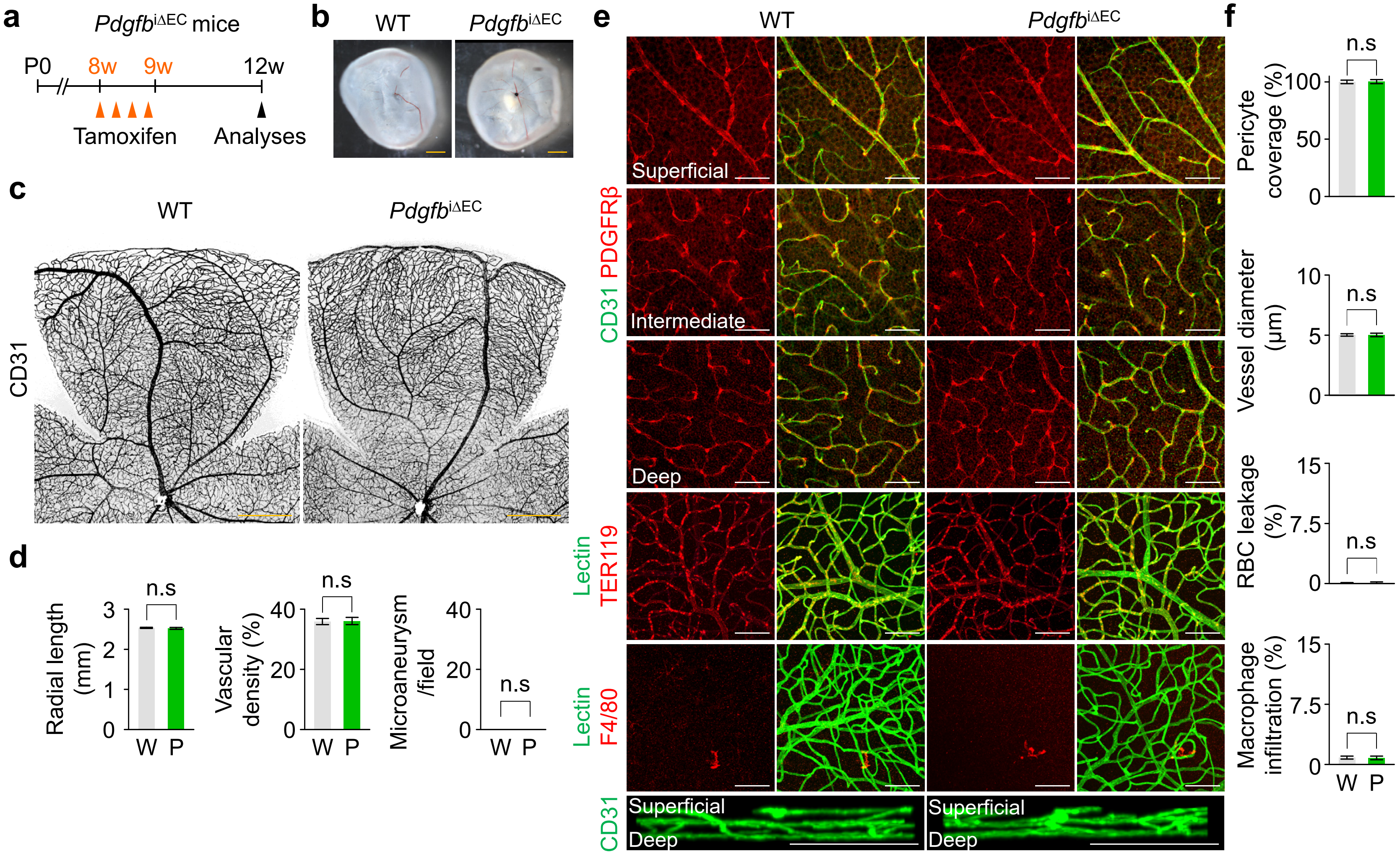

Supplementary Figure 12. PDGF-B/PDGFRβ signaling is not required to maintain EC-pericyte interaction for BRB integrity during adulthood. (**a**) Diagram depicting the experiment schedule for EC-specific depletion of PDGF-B in retinal vessels starting at 8 weeks after birth and their analyses at 4 weeks later using *Pdgfb*$^{i\Delta EC}$ mice. (**b-d**) Images of inner surface of retinal cup and CD31^{+} retinal vessels, and comparisons of indicated parameters in WT (W; *n* = 6) and *Pdgfb*$^{i\Delta EC}$ (P; *n* = 6) mice. (**e**) Images of CD31^{+} vessels and PDGFRβ^{+} pericyte coverage in the superficial, intermediate and deep vascular plexus, distribution of TER119^{+} RBC, F4/80^{+} macrophage infiltration, lectin perfusion, and cross-sectional images of retinal vascular plexus in W and P are shown. No significant differences were observed between the two groups. (**f**) Comparisons of indicated parameters in W (*n* = 6) and P (*n* = 6) mice. Error bars represent mean ± s.d. n.s., non-significant versus WT by Mann-Whitney *U* test. Scale bars, 100 μm (white) and 500 μm (yellow).

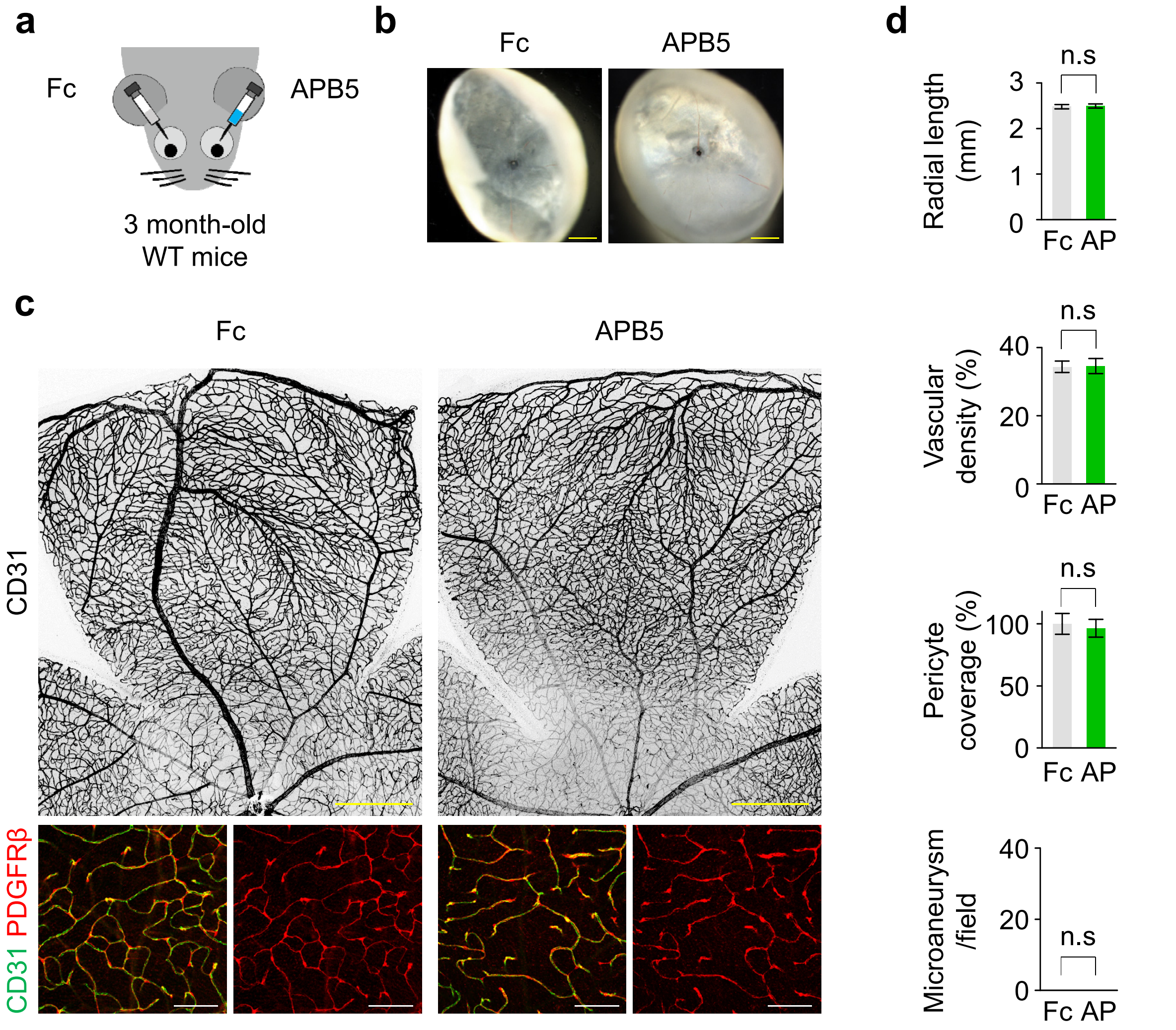

Supplementary Figure 13. No retinal vascular impairment upon intravitreal APB5 administration. (**a**) Administration of 5 μg of APB5 into vitreous cavity of one eye and Fc of contralateral eye in 3 month-old WT mice. Their retinas were analyzed 1 week after the intravitreal injection. (**b-d**) Images of inner surface of retinal cup and $CD31^+$ retinal vessels, $PDGFR\beta^+$ pericyte coverage and comparisons of indicated parameters in Fc treated (Fc; n = 4) and APB5 treated (AP; n = 4) eyes. Error bars represent mean ± s.d. n.s., non-significant versus Fc by Mann-Whitney U test. Scale bars, 100 μm (white) and 500 μm (yellow).

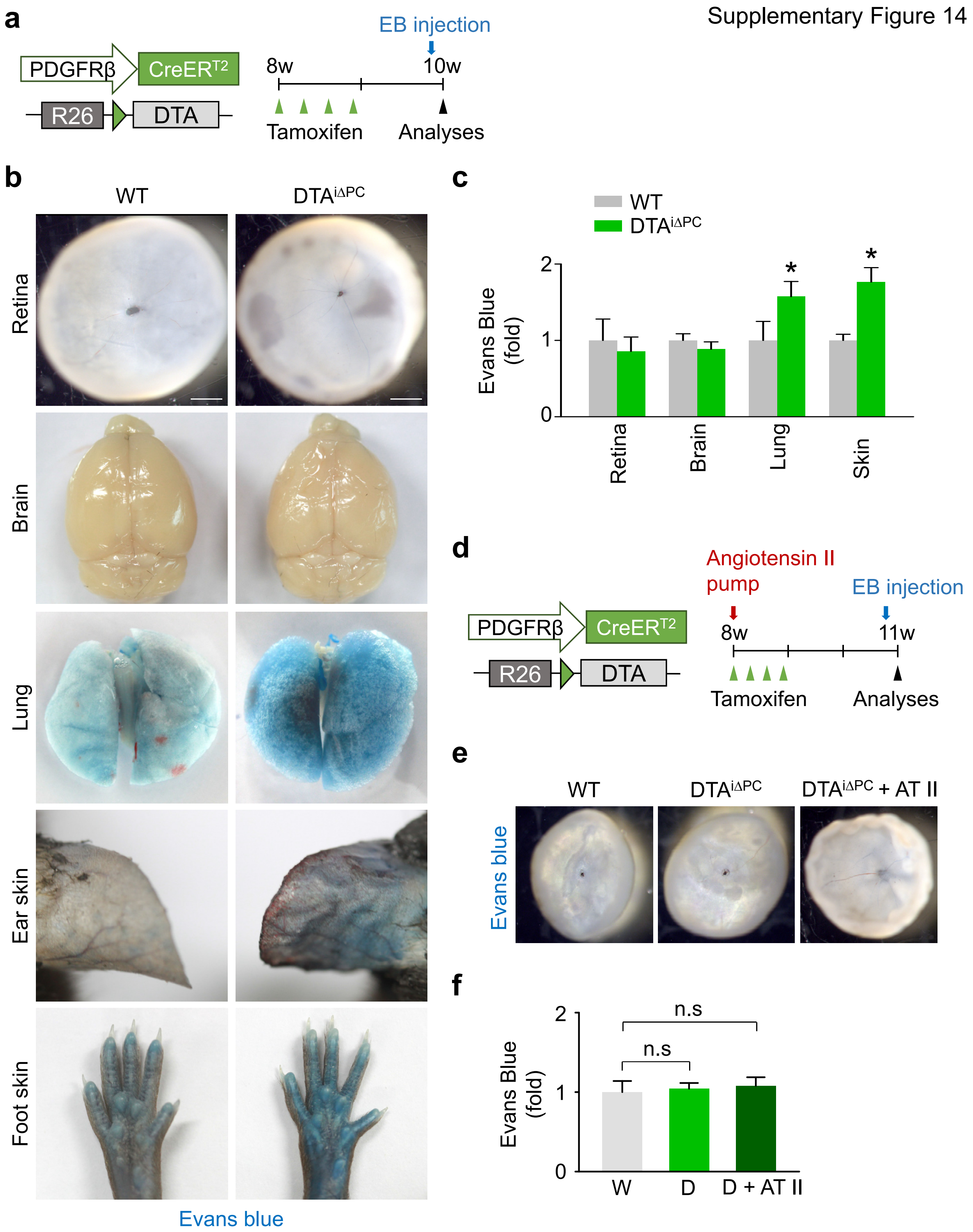

Supplementary Figure 14. Role of pericytes against vascular leakage is organ-specific during adulthood. (**a**) Diagram depicting the experiment schedule for selective loss of pericytes in 8-week-old DTA$^{i\Delta PC}$ mice and their analyses at 2 weeks later. Analysis of vascular leakage was performed by Evans blue (EB) injection. (**b,c**) Images and comparisons of inner surface of retina, brain, lung, and skins of ear and foot pad of WT and DTA$^{i\Delta PC}$ mice after EB injection are shown. Compared with WT mice, no apparent EB leakage is detected in retina and brain of DTA$^{i\Delta PC}$ mice, but profound EB leakage was observed in lung and skin of DTA$^{i\Delta PC}$ mice. Each group, $n = 4$. Error bars represent mean ± s.d. *$P < 0.05$ versus WT treated with EB by Mann-Whitney U test. (**d**) Diagram depicting the experimental schedule for selective loss of pericytes and implantation of angiotensin (AT) II osmotic pump in 8-week-old DTA$^{i\Delta PC}$ mice and their analyses at 3 weeks later. (**e,f**) Images of inner surface of retina and comparison of EB leakages in WT (W), DTA$^{i\Delta PC}$ mice (D), and DTA$^{i\Delta PC}$ mice with AT II pump (D + AT II). Each group, $n = 4$. Error bars represent mean ± s.d. n.s., non-significant versus WT by Kruskal-Wallis test. All scale bars, 500 μm (white).

Supplementary Figure 15

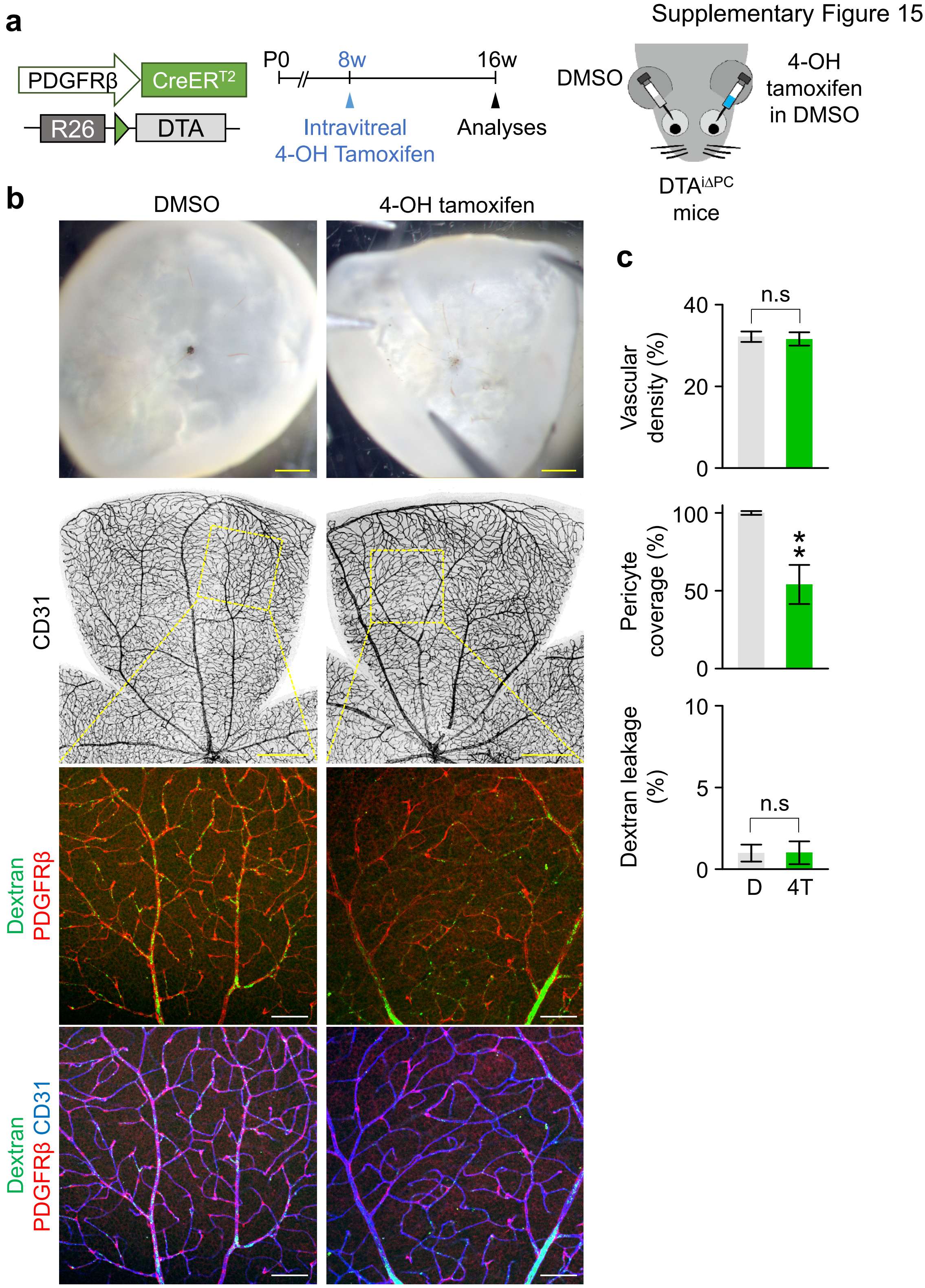

Supplementary Figure 15. Pericyte-free retinal vessels in adults are resistant to vascular leakage and disruption for a long-term period. (**a**) Diagram depicting the experiment schedule for selective loss of PC in retinal vessels of one eye by intra-vitreal injection of 4-OH tamoxifen in 8-week-old DTA$^{i\Delta PC}$ mice and their analyses at 8 weeks later. (**b,c**) Images and comparisons of inner surface of retinal cup, CD31^{+} vessels and PDGFRβ^{+} pericyte coverage around CD31^{+} superficial vascular plexus, and dextran (70 kDa) leakage in retinas administered with DMSO (D; *n* = 5) or 4-OH tamoxifen (4T; *n* = 5). Magnified images of boxed areas are shown below. Error bars represent mean ± s.d. **P* < 0.01, n.s., non-significant versus D by Mann-Whitney *U* test. Scale bars, 100 μm (white) and 500 μm (yellow).

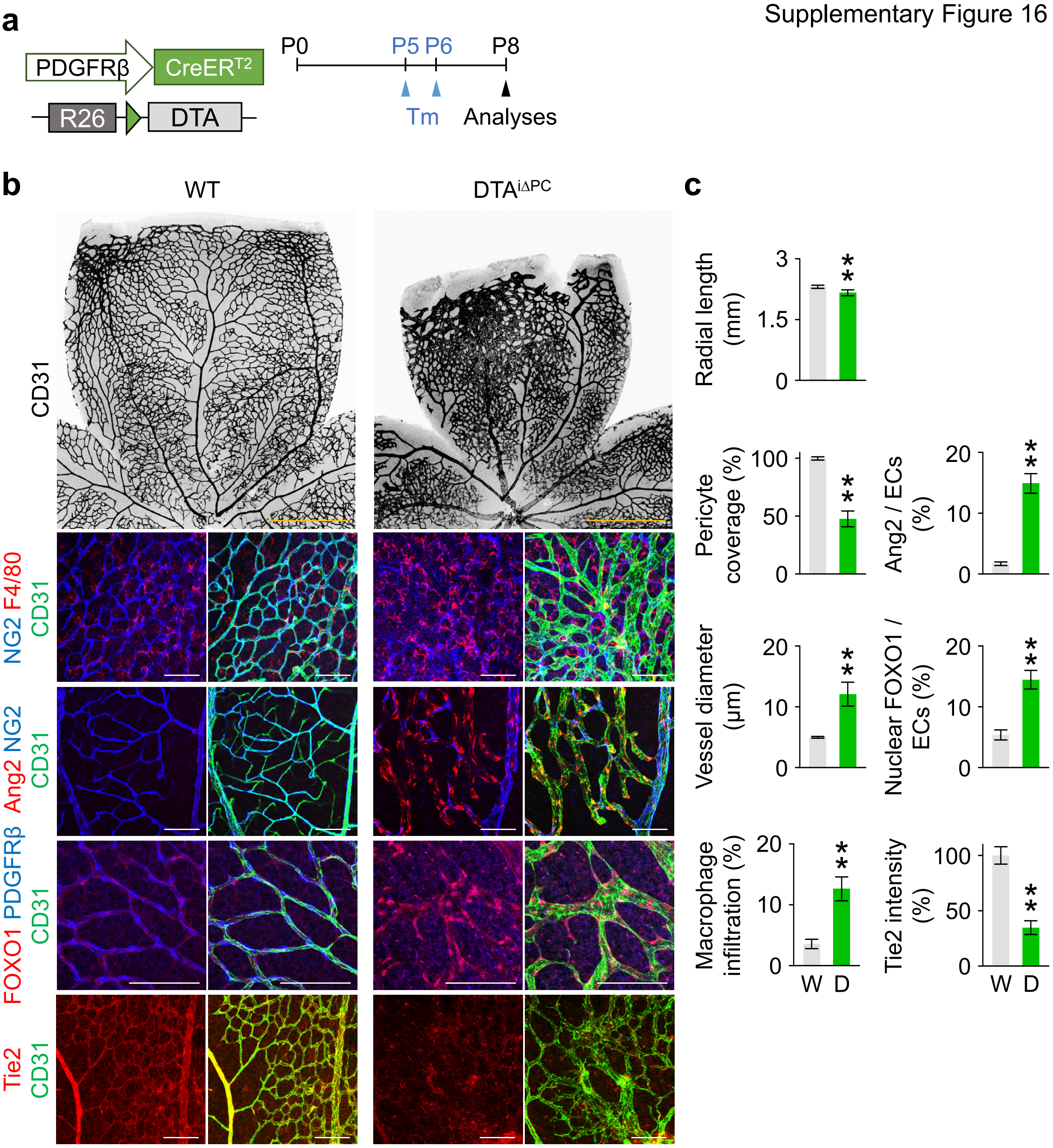

Supplementary Figure 16. Engorged retinal vessels by selective loss of pericytes during developmental period shows activation of FOXO1 and high expression of Ang2. (**a**) Diagram depicting the experiment schedule for selective loss of pericytes in retinal vessels from P5 and their analyses at P8 using DTA$^{i\Delta PC}$ mice. (**b**) Images of CD31^{+} vessels, NG2^{+} or PDGFRβ^{+} pericyte coverage, F4/80^{+} macrophage infiltration, and expression of Ang2, FOXO1, and Tie2 in the WT and DTA$^{i\Delta PC}$ mice. (**c**) Comparisons of indicated parameters in WT (W; *n* = 6) and DTA$^{i\Delta PC}$ (D; *n* = 6) mice. Error bars represent mean ± s.d. $^{**}P < 0.01$ versus WT by Mann-Whitney *U* test. n.s., non-significant. Scale bars, 100 μm (white) and 500 μm (yellow).

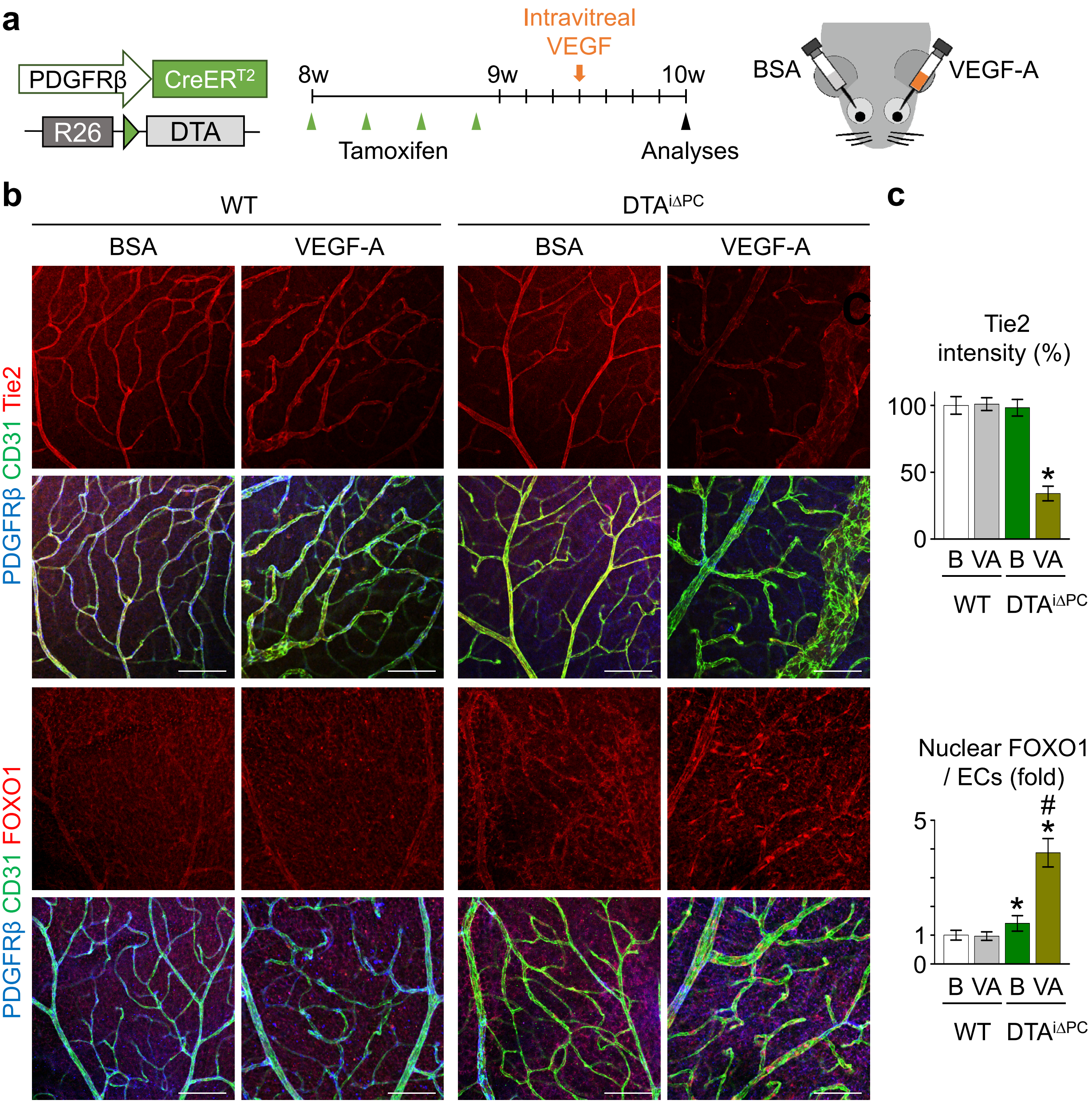

Supplementary Figure 17. Additional insult with excessive VEGF-A induces Tie2 down-regulation and FOXO1 activation in the pericyte-free retinal vessels of adult mice. (**a**) Diagram depicting the experiment schedule for selective loss of pericytes in retinal vessels, intra-vitreal administration of VEGF-A (1 μg) into one eye and BSA (1 μg) into the contralateral eye, and analyses that were performed at 4 days after VEGF-A administration using adult $DTA^{i\Delta PC}$ mice. (**b,c**) Images and comparisons of Tie2 and FOXO1 expression by $CD31^{+}$ vessels in retinas of WT and $DTA^{i\Delta PC}$ mice treated with BSA (B) or VEGF-A (VA). Each group, $n = 6$. Error bars represent mean ± s.d. $^{*}P < 0.01$ versus WT treated with B, $^{\#}P < 0.05$ versus $DTA^{i\Delta PC}$ treated with B by Kruskal-Wallis test. All scale bars, 100 μm (white).

Supplementary Table 1. Primer sets for quantitative real-time RT-PCR for mouse samples.

Name	Sequence (5'-3')	
CCL-2	Forward	CCCAATGAGTAGGCTGGAGA
	Reverse	AAAATGGATCCACACCTTGC
CCL-3	Forward	ACTGCCTGCTGCTTCTCCTACA
	Reverse	AGGAAAATGACACCTGGCTGG
TNF-α	Forward	CACGCTCTTCTGTCTACTGAACTTCG
	Reverse	GTGGGCTACAGGCTTGTCACTC
Arginase-1	Forward	CAGAAGAATGGAAGAGTCAG
	Reverse	CAGATATGCAGGGAGTCACC
Arginase-2	Forward	TGATTGGCAAAAGGCAGAGG
	Reverse	CTAGGAGTAGGAAGGTGGTC
IL-10	Forward	GCTCTTACTGACTGGCATGAG
	Reverse	CGCAGCTCTAGGAGCATGTG
IL-6	Forward	CGGAGGCTTAATTACACATGTTCTCTGG
	Reverse	CCAGGTAGCTATGGTACTCCAGAAGAC
VEGF-A	Forward	CTCCACCATGCCAAGTGGTC
	Reverse	TCGTTACAGCAGCCTGCACA
Tie2	Forward	ACGGACCATGAAGATGCGTCAACAA
	Reverse	TCACATCTCCGAACAATCAGCCTGG
GAPDH	Forward	TGTTCCTACCCCCAATGTGT
	Reverse	TGTGAGGGAGATGCTCAGTG

Supplementary Table 2. Primer sets for quantitative real-time RT-PCR for human samples.

Name	Sequence (5'-3')	
Ang2	Forward	TGCCACGGTGAATAATTCAG
	Reverse	TTCTTCTTTAGCAACAGTGGG
Tie2	Forward	TTGAAGTGGAGAGAAGGTCTG
	Reverse	GTTGACTCTAGCTCGGACCAC
ESM1	Forward	TGGCCGCCTGGAGCAATAA
	Reverse	TCCTCCCCATTAGAAGGCTGACA
GAPDH	Forward	GGTGGTCTCCTCTGACTTCAACA
	Reverse	GTTGCTGTAGCCAAATTCGTTGT

Supplementary Table 3. Primers used in ChIP-qPCR.

Human Hg19, Chromosome 8

	Start	End	Forward primer	Reverse primer
1	6,457,902	6,456,259	GCAGTCCCTGACTAACTCCG	CGCGGACCGGAAGGATATTT
2	6,435,742	6,433,956	CTGGCCACCTGGATTAACGA	TCCTGCCACTGGAATTACGC
3	6,420,792	6,415,306	TTGGCCGCAGCCTATAACAA	ACAGCATTGGACACGTAGGG
4	6,406,146	6,404,350	GTGACGCACGAGTAGAGTCC	GCGGCACGCTTTGACTTTAG
5	6,387,041	6,385,569	TCTTCCTGCTGAAGGGCAAC	CGAAGCCGTATAGCGTGAGT
6	6,369,127	6,367,185	TAGAGACTGAGCTACGGCCA	GAGGGTAGGGCCATGTGATG
7	6,345,581	6,343,691	TAGGAAGCTCGGCAACTCCT	CAGCAGTTCACACCTCCAGT
